# Assessing the impact of oral health disease on quality of life in Ecuador: a mixed-methods study

**DOI:** 10.3389/froh.2024.1431726

**Published:** 2024-07-18

**Authors:** Nupur Krishnan, Karem Manresa-Cumarin, Jessica Klabak, Greg Krupa, Priyanka Gudsoorkar

**Affiliations:** ^1^ Michael G. DeGroote School of Medicine, McMaster University, Hamilton, ON, Canada; ^2^ Department of Epidemiology and Biostatistics, University of Illinois at Chicago School of Public Health, Chicago, IL, United States; ^3^ Solidarity Dental Foundation, Quito, Ecuador; ^4^ College of Medicine, University of Cincinnati, Cincinnati, OH, United States

**Keywords:** oral health quality of life, oral health disparities, oral health promotion, oral health disease, social determinants of health, barriers to oral health

## Abstract

**Introduction:**

Globally, oral health diseases surpass all other non-communicable diseases in prevalence; however, they are not well studied in underserved regions, where accessibility to dental services and oral health education is disparately worse. In Ecuador, further research is needed to understand such disparities better. We aimed to assess the effect of oral health disease on individuals' quality of life and how social disparities and cultural beliefs shape this.

**Methods:**

Individuals 18 or older receiving care at mobile or worksite clinics from May to October 2023 were included. A mixed-methods approach was employed, involving semi-structured interviews, Oral Health-Related Quality of Life (OHRQoL) measures, and extra-oral photographs (EOP).

**Results:**

The sample (*n* = 528) included mostly females (56.25%) with a mean age of 34.4 ± 9.44. Most participants (88.26%) reported brushing at least twice daily, and less than 5% reported flossing at least once per day. The median OHRQoL score was 4 (min-max), significantly higher among individuals ≥40 years old, holding high school degrees, or not brushing or flossing regularly (*p* < 0.05). Identified barriers to good oral health included affordability, time, and forgetfulness. Participants not receiving care with a consistent provider reported fear as an additional barrier. Participants receiving worksite dental services reported these barriers to be alleviated. Dental providers were the primary source of oral hygiene education. Most participants reported oral health concerns, most commonly pain, decay, dysphagia, and halitosis - consistent with EOP analysis.

**Discussion:**

Findings underscore a need for multi-level interventions to advance oral health equity.

## Introduction

Throughout their lives, almost half of the global population is affected by oral disease (1). The global number of oral disease cases is estimated to exceed that of the next five most common noncommunicable diseases combined (1). These rates, however, are not uniformly distributed worldwide, with just under 85 percent of the individuals affected by oral disease coming from low- or middle-income countries (LMICs) (1). Additionally, various social, economic, and political determinants in these countries contribute to further oral health inequities. For instance, disparities in accessibility, availability, and affordability of oral health care can be seen across factors such as income, socioeconomic status, education level, gender, and remote or rural communities (1–3). Individuals facing such disparities have been identified as some of the most vulnerable and have been found, on average, to have fewer teeth than individuals who have more resources or better access to care (1). It has also been seen globally that unique religious or traditional practices, lifestyle factors, and cultural beliefs have the potential to contribute to deteriorating oral health (1–4). Such trends in oral disease affect individuals' quality of life, with consequences such as pain, discomfort, functional limitations, compromised nutrition and growth, poorer attendance at school or work, decreased self-esteem, and impacts on general health (1). Moreover, the adverse effects extend further, as oral disease presents a considerable burden to health systems, with an estimated $298 billion USD being spent on direct oral disease treatment costs globally each year (1, 5).

Like many other LMICs, Ecuador grapples with disparities in oral health that impact diverse age groups and socio-economic strata. Almost half (45%) of children 9 years of age and under have untreated caries in deciduous teeth, and in 2019, the estimated total productivity losses due to the combined impact of various oral health diseases were measured at $417 million USD (6). Clearly, oral disease is a concern that warrants further attention.

In addition to the rising trend of oral diseases globally, and in Ecuador, it is becoming apparent that there is a lack of comprehensive oral health research and data. At the 2021 World Health Assembly, the World Health Organization released a resolution on oral health, highlighting the need for improved oral health data to develop oral health promotion strategies and address oral health inequalities worldwide (7). In this statement, they highlighted the need for a better understanding of determinants of oral health inequities and risk factors for disease to develop interventions and policies to address them (7). Rural and remote regions have been identified as the most underserved and understudied regions, as is also the case in Ecuador (7).

We aimed to assess the effect of oral health disease on individuals' quality of life in Ecuador, as well as to understand how this is shaped by social disparities and cultural beliefs. This research will inform evidence-based strategies to enhance oral health outcomes, expand access to dental care, and ultimately improve public health in Ecuador.

## Methods

### Data collection process

Novulismed SA (Novulis) is a social enterprise that provides affordable, high-quality onsite dental care and education to underserved communities through mobile and stationary clinics. We included male and female patients aged 18 or older who received dental care at Novulis locations across Ecuador from May to October of 2023.

The recruitment of participants was done using purposive sampling. After obtaining informed consent (See Supplementary Table S1. Consent Form), each participant received a unique 6-digit identifier (e.g., BF.03.01). Demographic information as well as self-reported oral hygiene habits were collected via paper questionnaire. The Oral Health-Related Quality of Life (OHRQoL) survey was administered to participants in paper-pencil format (See Supplementary Table S2. Demographic Questionnaire & OHRQoL measure). The OHRQoL survey consisted of a 5-point Likert scale that reflected participants' self-perceived impact of oral health status on three dimensions of their quality of life: affectation of daily activities, social activities, and conversation abilities. The OHRQoL survey, in the form of a Likert scale, has been used in previous literature (8, 9). For our study, it was translated into Spanish and adapted for cultural nuances/understanding with the assistance of local translators.

A subset of the sample was then recruited based on theoretical saturation (*n *= 36) to perform a qualitative assessment consisting of an extraoral photograph and semi-structured interview. Following consent, an extraoral photograph was taken and saved as a digital file labelled with the participant's assigned 6-digit identifier to facilitate matching with other records from the same participant. The photograph was taken with a standard encrypted memory card with a point-and-click camera and captured from the base of the nose to the tip of the chin, smiling, frontal view. After the photo was accurately saved and marked, the qualitative interview began with the aid of a language interpreter. A semi-structured interview guide was used to comprehensively understand oral hygiene practices and participants' current oral health status (See Supplementary Table S3. Semi-structured Interview Guide). This guide was previously piloted for comprehension and cultural suitability (10). The interviews lasted between 30 and 45 min and the interviewer verbally stated the participant's assigned 6-digit identifier on the audio recording at the beginning of each interview to facilitate record matching. Each interview was conducted at a mutually agreed upon site: a mobile or stationary Novulis worksite dental clinic. Field notes, extraoral photographs, interview responses, and OHRQoL scores were matched and compared using the participants' unique identifiers.

### Quantitative data analysis process

Continuous variables of age and income were divided into categories. Age was dichotomized using 40 years as the cut-off point, and income categories were defined based on a local minimum wage of $450 USD. Behavioral variables were categorized as follows: brushing teeth at least twice per day vs. less; flossing at least once per day vs. less; smoking tobacco/vaping at least once a day vs. not; and having at least one drink once a week vs. not.

The descriptive analysis focused on demographic and clinical measures. An overall OHRQoL score was obtained as a sum of scores derived from the responses (3–15; min-max), with higher scores indicating decreased OHRQoL. Median scores for each question of the OHRQoL questionnaire were reported. Descriptive statistics were calculated, stratified analysis was carried out, and frequencies of responses were reported. F-test of homogeneity, Wilcoxon rank sum test, and ANOVA were used to test variance differences between groups. The quantitative data were analyzed using RStudio statistical software 2023, version 12.1+402, and significance was established at *p* < 0.05.

### Qualitative data analysis process

Each interview was transcribed and read twice by three researchers independently to become immersed in the data. Inductive reasoning and thematic analysis were used to identify codes. Memos were created on MAXQDA 2022, Version 22.1.1, and were concurrently referred to throughout the coding procedure for context. Similar codes were combined into like categories and later condensed into five overarching themes. Criteria used to develop themes included a key phrase or combination of significant code categories well-represented in the data. A constant comparison method was utilized throughout the qualitative analysis in two stages: comparing incidents applicable to each category and integrating categories and their properties. This allowed for identifying relationships between the emerging themes. Exemplar quotes were identified to supplement the findings' description and maintain the themes' authenticity.

Extra-oral photographs were examined for both dental and facial characteristics. Qualitative analysis of photographs included a thematic analysis of three predefined categories: hard tissue, soft tissue, and smile. The hard tissue theme described conditions of the teeth and mouth and included assessing for discoloration, fractures, attrition, abrasion, missing teeth, and presence of plaque; soft tissue described characteristics of the gums, lips, and labial mucosa and included features such as discolorations, inflammation, lesions, gingival recession, and dryness of mucosa and lips; smile analysis included the anatomic smile classification, presence of gummy smile, and alignment of lips.

## Results

### Participants and demographics

A total of 528 participants were included in the sample (Table 1). Most participants identified as female (*n *= 297, 56.25%). The average reported age was 34 years (*SD* ± 9.44 range: 18–69), and 69% of the sample (*n *= 365) were under 40 years old. Regarding education level, just over half of the sample (*n *= 296, 56%) reported having at least a high school diploma. The mean reported household income was $669 USD (*SD* ± 418.53; range: $125–3,600 USD) per month (Table 1). Overall, participants reported being residents of 17 provinces (out of Ecuador's 24), most of whom resided in the Pichincha (43.5%, *n* = 230), Cotopaxi (23.3%, *n* = 123), and Imbabura (20.6%, *n* = 109) provinces, all of which belong to the inter-Andean region of La Sierra.

**Table 1:** Participant characteristics.

Variable	Total 528 (100%)	Mean ± SD	Median ± IQR	Missing *n* (%)
Demographic				
Sex		–	–	0 (0%)
Females	297 (56.25%)			
Males	231 (43%)			
Age groups		34.4 ± 9.44	34 ± 15	0 (0%)
<40	365 (69.13%)			
≥40	163 (30.87)			
Education level		–	–	0 (0%)
Less than a high school degree	232 (43.94%)			
At least high school degree	296 (56.06%)			
Household income		669 ± 418.53	500 ± 83	2 (0.38%)
<450	31 (5.87%)			
450–900	406 (76.89%)			
>900	90 (17.04%)			
Behavioral				
Brushing frequency		–	–	0 (0%)
Less than 2 per day	62 (11.74%)			
At least 2 per day	466 (88.26%)			
Flossing Frequency		–	–	0 (0%)
Less than 1 per day	509 (96.4%)			
At least 1 per day	19 (3.60%)			
Regular smoker	244 (46.21%)	–	–	0 (0%)
Regular alcohol consumer	236 (44.70%)	–	–	0 (0%)
OHRQoL	–	–	4 ± 3	31 (5.87%)
Question 1	–	–	1 ± 1	
Question 2	–	–	1 ± 1	
Question 3	–	–	1 ± 1	

### Quantitative findings

Regarding oral hygiene practices (Table 1), 88.26% (*n *= 466) reported brushing their teeth at least twice per day, but only 3.6% (*n* = 19) reported using dental floss at least once daily. The majority (96.4%; *n *= 509) reported either not using dental floss at all or, if at all, very sporadically. When surveyed, 46.21% (*n *= 244) of participants reported being regular users of smoking tobacco or vapes, and 44.7% (*n *= 236) reported being regular alcohol consumers.

### Oral Health-Related Quality of Life (OHRQoL)

**Figure 1:**
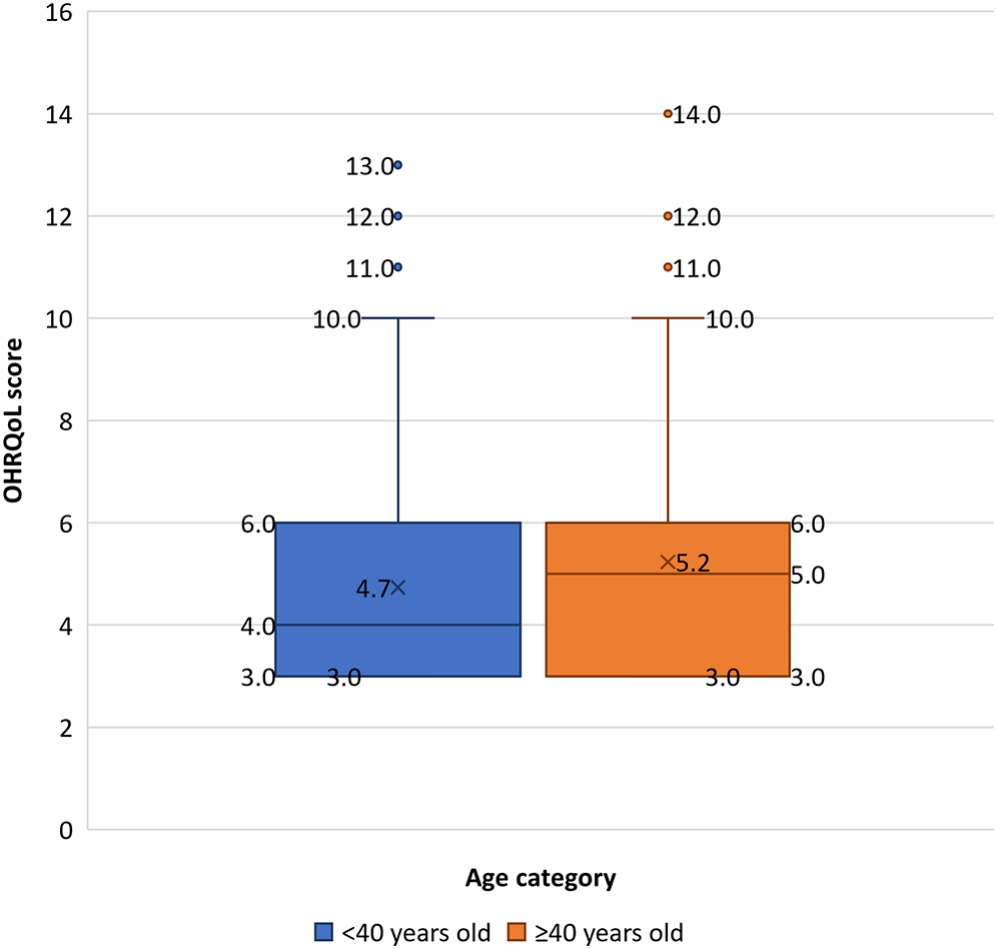
Boxplot showing OHRQoL score distribution across age categories. Blue indicates participants aged <40 years; orange indicates participants aged ≥40. Shaded box refers to the interquartile range; horizontal line through box indicates median; and “X” depicts mean. Upper and lower whiskers indicate maximum and minimum respectively. Individual points outside the whiskers represent outliers.

**Figure 2:**
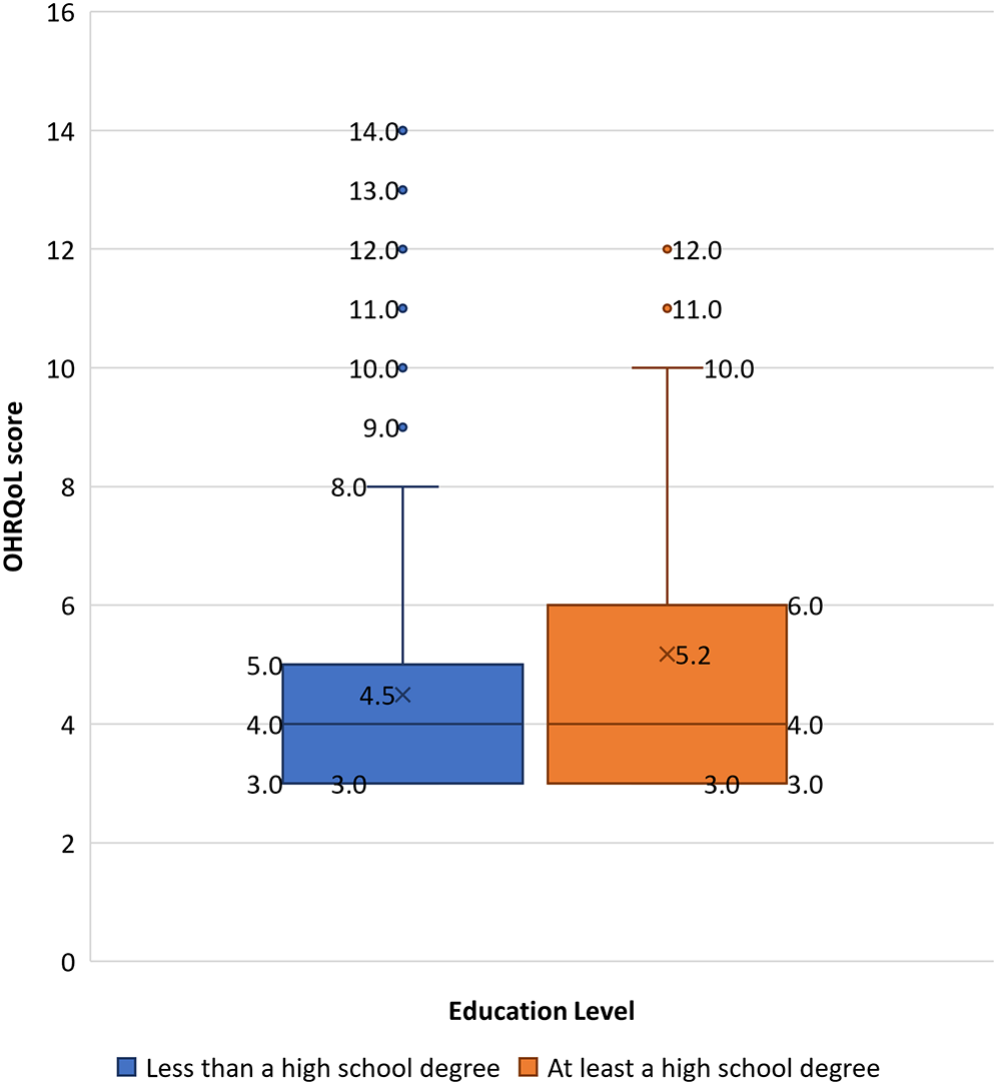
Boxplot showing OHRQoL score distribution across education level categories. Blue indicates participants who have less than a high school degree; orange indicates participants who have at least a high school degree. Shaded box refers to the interquartile range; horizontal line through box indicates median; and “X” depicts mean. Upper and lower whiskers indicate maximum and minimum respectively. Individual points outside the whiskers represent outliers.

**Figure 3:**
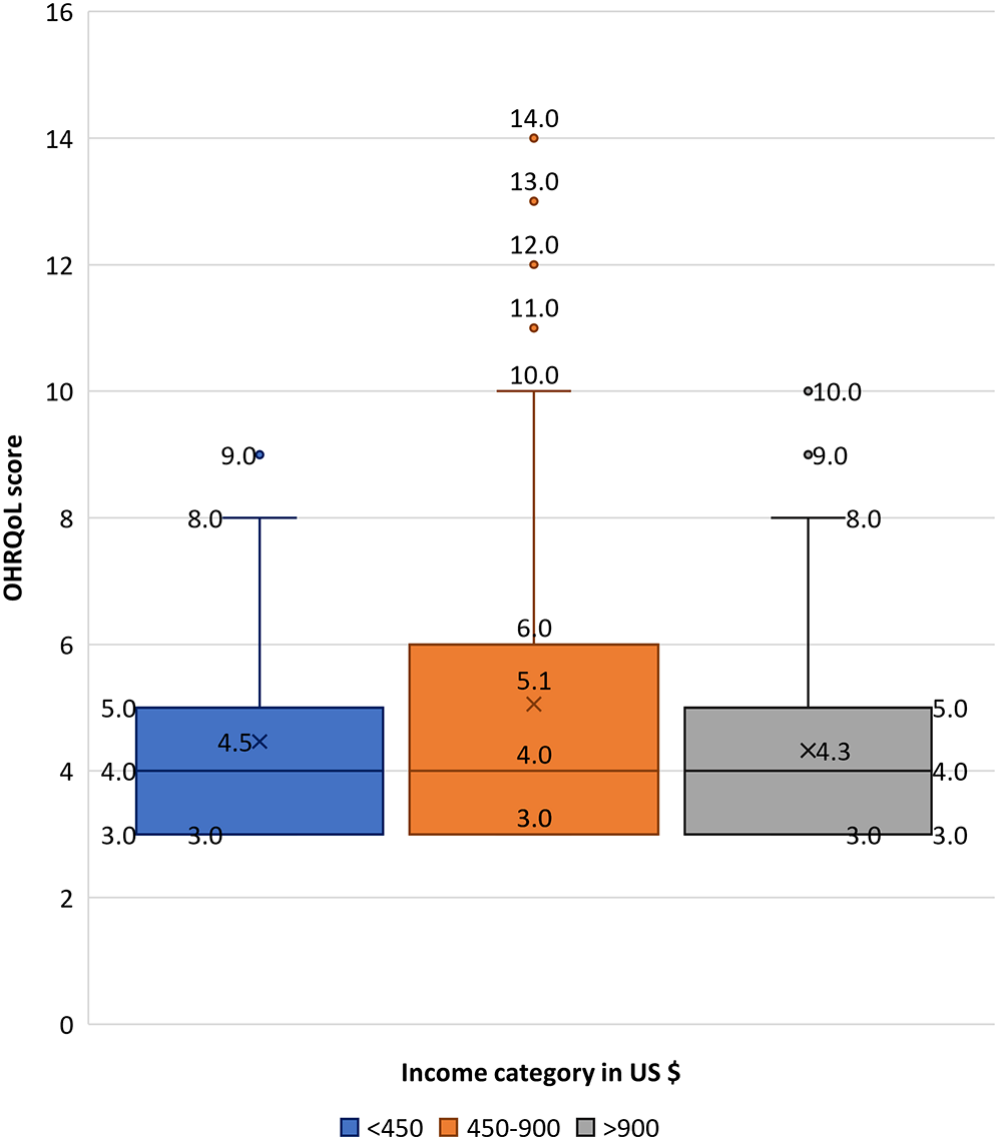
Boxplot showing OHRQoL score distribution across income categories. Blue indicates participants with a household income of <450 USD; orange indicates a household income of 450–900 USD, and grey indicates a household income of >900 USD. Shaded box refers to the interquartile range; horizontal line through box indicates median; and “X” depicts mean. Upper and lower whiskers indicate maximum and minimum respectively. Individual points outside the whiskers represent outliers.

All participants (*n *= 528) consented to the OHRQoL measure. After removing incomplete records, the OHRQoL was analyzed for 497 participants. The overall sample reported a median OHRQoL score of 4 (IQR* *± 3; range: 3–14) (Table 1), with scores evenly distributed across all three questions. Bivariate analysis (Table 2) revealed that the OHRQoL scores were evenly distributed across sex, household income categories, smoking status, or alcohol consumption. Individuals aged 40 years or greater had significantly higher median OHRQoL scores (5 ± 3) compared to younger participants (4 ± 3) (*p *< 0.05) (Figure 1). The density of distribution of OHRQoL scores was significantly higher among individuals who had at least a high school degree compared to those who had not completed high school (*p *< 0.05) (Figure 2). Those who brushed at least twice per day had significantly lower OHRQoL scores (4 ± 3) compared to those who did not (5 ± 4) (*p *< 0.05). A similar trend was seen regarding flossing frequency (3 ± 0 vs. 4 ± 3 respectively) (*p *< 0.05). While insignificant, a trend was observed in which those with an income between $450–900 USD showed a higher third quartile and higher range of OHRQoL scores than the other two income brackets (Figure 3).

**Table 2:** Mean OHRQoL scores and standard deviations by independent variables.

Variable	OHRQoL Mean ± SD	OHRQoL Median ± IQR	*p*-value
Demographic			
Sex			0.1529
Females	4.81 ± 2.09	4 ± 3	
Males	4.99 ± 2.56	4 ± 3	
Age groups			0.0125
<40	4.74 ± 2.23	4 ± 3	
≥40	5.24 ± 2.49	5 ± 3	
Education level			0.0002
Less than a high school degree	4.49 ± 2.26	4 ± 2	
At least high school degree	5.18 ± 2.33	4 ± 3	
Household income ($ USD)			
<450	4.47 ± 1.61	4 ± 2	0.6767
450–900 (Reference)	5.06 ± 2.49	4 ± 3	
>900	4.33 ± 1.59	4 ± 2	0.0166
Behavioral			
Brushing frequency			0.0029
Less than 2 per day	5.59 ± 2.57	5 ± 4	
At least 2 per day (Reference)	4.79 ± 2.27	4 ± 3	
Flossing frequency			0.0009
Less than 1 per day	4.94 ± 2.33	4 ± 3	
At least 1 per day (Reference)	3.68 ± 1.70	3 ± 0	
Regular smoker	4.92 ± 2.47	4 ± 3	0.8672
Regular alcohol consumer	4.96 ± 2.48	4 ± 3	0.8068

### Qualitative findings

A subset of 36 participants was randomly selected for involvement in further qualitative analysis (semi-structured interviews and extra-oral photographs). Most participants were female (*n *= 28; 77.78%) with a mean age of 37.69 (SD* *± 11.07) (Table 3). Over half of the sample reported having at least a high school degree (*n *= 20; 55.56%), and the mean reported household income was $669 USD (SD* *± 418.53). Participants in this subset reported being residents of 3 provinces, of which 61% (*n* = 22) resided in the province of Pichincha. The median OHRQoL index of the subset was 4 (IQR 2.5).

**Table 3:** Patient characteristics of the qualitative sample subset.

Variable	Total 36 (100%)	Mean ± SD	Median ± IQR	Missing *n* (%)
Demographic				
Sex				0 (0%)
Females	28 (77.78%)	–	–	
Males	8 (22.22%)	–	–	
Age groups		37.69 ± 11.07	–	0 (0%)
<40	19 (52.78%)			
≥40	17 (47.22)			
Education level		–	–	1 (2.78%)
Less than a high school degree	15 (41.67%)			
At least high school degree	20 (55.56%)			
Household income ($ USD)		669 ± 418.53	475 ± 102	9 (25.00%)
<450	7 (19.44%)			
450–900 (Reference)	20 (55.56%)			
>900	0 (0.00%)			
Behavioral				
Brushing frequency		–	–	0 (0%)
Less than 2 per day	11 (30.56%)			
At least 2 per day	25 (69.44%)			
Flossing frequency		–	–	0 (0%)
Less than 1 per day	26 (72.22%)			
At least 1 per day	10 (27.78%)			
Regular smoker	1 (2.78%)	–	–	5 (13.89%)
Regular alcohol consumer	4 (11.11%)	–	–	5 (13.89%)
OHRQoL		–	4.00 ± 2.50	0 (0.00%)
Question 1	–	–	1 ± 1	
Question 2	–	–	1 ± 0.50	
Question 3	–	–	1 ± 1	

### Thematic analysis

The initial theme, Oral Hygiene Routines & Habits, reflected that 25 participants (69.44%) (Table 3) reported brushing at least twice per day and understood the importance of oral health as a routine practice to be incorporated into daily life. However, this still leaves about one-third of the participants (*n *= 11, 30.56%) who reported inconsistent tooth-brushing practices. A few reasons for the inconsistency included lack of accessibility and affordability of dental hygiene aids (*n *= 3), lack of time (*n *= 2), and forgetfulness (*n *= 5). For example, one participant said, “*I have to rush to work in the morning, so I do not get time to brush, and because I’m so tired at night, I forget to brush.”* Another participant stated, “*Our family is really big, so sometimes I cannot always have a toothbrush of my own”* (Table 4). A suboptimal flossing frequency was evident among most respondents, and only 10 participants (27.78%) reported regular flossing.

**Table 4:** Descriptions and definitions of five qualitative themes.

Theme	Definition	Sample quotes
Oral hygiene routines and habits	Describes participants’ oral hygiene behaviours, including tooth brushing frequency, flossing and mouthwash habits, alternate cleaning methods such as use of natural products/ substitutions, and other related routines.	“I have to rush to work in the morning, so I do not get time to brush, and because I’m so tired at night, I forget to brush.” “Our family is really big, so sometimes I cannot always have a toothbrush of my own” “I do not know we were supposed to clean the tongue.” “The family and I do not use anything else to brush our teeth except toothbrush and toothpaste.”
Knowledge acquisition and education	This theme examines participant reports about the origins of oral hygiene knowledge, such as through family, school programs, or professional dental care, or a lack of education at all. It also covers intergenerational transmission of oral hygiene education.	“My parents started brushing my teeth for me when I was really little and made sure I did it myself twice a day once I was old enough. My mom would check in the mornings, but never before bed, so I don't brush at night” “The dentist showed me models of how food and plaque can get stuck between teeth and let me practice brushing and once showed how to get flossing done on a large model mouth. I was motivated to brush but still cannot afford the floss each time.” “I started brushing my own teeth at 12 years old. I am from [an Indigenous area], where they don't have this custom of brushing their teeth. …. But, when I was 12, I went to live in Bogota, Colombia, and the people I lived with taught me that I needed to brush my teeth.”
Barriers to oral hygiene and dental care	This theme analyzes participant-reported challenges to maintaining oral hygiene and accessing professional dental care, including constraints such as time, cost, transportation, accountability, and external factors like the COVID-19 pandemic.	“During the pandemic, we didn't have toothpaste and were having trouble getting it. So for 4 months, we just used the toothbrush alone, without any toothpaste.” “When I was 12, I was scared. I had a cavity, and they made it like a hole, like a huge hole in the tooth, and like put the patch in it. It was a horrible experience because I could not talk or eat anything for a long time. So, when I grew up, I never attended the checkups. I’m just scared” “I really appreciate the convenience of having dental services available on-site at my workplace. It saves me time and hassle, and I can easily fit appointments into my schedule.”
Diet influences on oral health	This theme investigates dietary and lifestyle factors influencing oral health, such as sugar consumption as well as tobacco and alcohol use.	“Everybody in the family really likes sweets. We all eat candies at least once a day.” “If you eat too much of the sugary foods, then it's harmful to your teeth, but if you eat a few during the day, but then you brush your teeth afterward, then it will be fine.” “Occasionally, I allow my children to have two servings of juice [including Pepsi and Coke]. When I notice that they drink the juice quickly, I think they are thirsty. In such cases, I say, ‘You can have another one if you want to.”
Self- reported Oral Health Status	This theme identifies participant-reported oral health issues, including pain, decay, swelling, and halitosis.	“I couldn't ignore the constant, throbbing ache in my tooth that night. It was like a sharp reminder that dental pain is not something to be taken lightly.” “I’ve never been good about taking my children to the dentist, and honestly, I don't go myself unless it hurts.”

Additionally, tongue cleaning was found to be a less widely held habit or not practiced at-all. One participant commented, “*I do not know we were supposed to clean the tongue.”* Most respondents (*n *= 22, 61.11%) reported using mouthwash; however, when further asked about this practice, participants shared that their use was inconsistent. When reflecting on overall hygiene practices, one participant said, “*The family and I do not use anything else to brush our teeth except toothbrush and toothpaste,”* demonstrating room for improvement.

The second theme, Knowledge Acquisition & Education, demonstrated the role of early and consistent oral hygiene education through family and institutional influences. Over one-quarter (*n *= 10; 27.78%) of adults reported strongly influencing children's brushing habits. For example, one participant stated, “*I started brushing my kid's teeth at six because my mom explained that she brushed my teeth at six.”* Another participant shared, “*My parents started brushing my teeth for me when I was little and made sure I did it myself twice a day once I was old enough. My mom would check in the mornings, but never before bed, so I don't brush at night”*. Some parents also reported prioritizing their children's oral health education and care over their own, as evidenced by the following quotation, “*I am a grown-up so there is nothing you can do about my teeth, but my son can start having great oral health.”* While peers and siblings have a relatively minor reported influence (*n *= 2; 5.56%), dental providers were reported to have the strongest influence (*n *= 17; 47.22%). For example, one participant stated, “*The dentist gave me a new toothbrush and had me practice circular scrubbing motions on a model set of teeth. She gave feedback like -Make sure you get the backs of the teeth too!”* and another stated, “*The dentist showed me models of how food and plaque can get stuck between teeth and let me practice brushing and once showed how to get flossing done on a large model mouth. I was motivated to brush but still cannot afford the floss each time.”* Oral hygiene education was also found to be influenced by social and cultural beliefs and practices. One participant shared “*I started brushing my teeth at 12 years old. I am from [an Indigenous area], where they don't have this custom of brushing their teeth. …. But, when I was 12, I went to live in Bogota, Colombia, and the people I lived with taught me that I needed to brush my teeth.”*

The third theme, Barriers to Oral Hygiene and Dental Care, highlights the challenges posed by the cost and availability of hygiene products and professional dental care, especially during the COVID-19 pandemic. One participant expressed, “*During the pandemic, we didn't have toothpaste and were having trouble getting it. So, for 4 months, we just used the toothbrush alone, without any toothpaste.”* As healthcare services resumed following the COVID-19-precipitated lockdowns, several respondents (*n *= 12; 33%) reported that worksite dental care helped alleviate barriers to access like transportation, time away from work, and cost. One participant expressed, “*I appreciate the convenience of having dental services available on-site at my workplace. It saves me time and hassle, and I can easily fit appointments into my schedule.”*

Additionally, a few (*n* = 2; 5.56%) shared that having worksite dental care services allows them to choose multi-visit treatment options like endodontics and orthodontics. One participant stated, “*When [the onsite] orthodontic program helped me get braces, it changed my smile and my confidence. I used to hide my teeth when laughing because I was embarrassed. Now I smile big and proud, thanks to getting the care I needed but couldn't afford to travel for each appointment before.”* Another participant said, “*I never thought straight, beautiful teeth were possible for someone like me, but now I can't stop grinning because I finally will have the smile I deserve despite my circumstances.”*

Participants who were not receiving regular dental care with a consistent provider reported fear of dental visits (*n *= 5) and expressed that this was a barrier to seeking dental care. A participant reported, “*When I was 12, I was scared. I had a cavity, and they made it like a hole, like a huge hole in the tooth, and like put the patch in it. It was a horrible experience because I could not talk or eat anything for a long time. So, when I grew up, I never attended the check-ups. I’m just scared”*.

Conversely, several participants (*n *= 14) who received regular care, for example, through worksite dental clinics, reported that consistent professional dental care helped them overcome the fear and anxiety of dental visits. For example, one participant stated, “*So the main difference in the service is that they have patience here; they do everything gently. A co-worker told me they put on anesthesia when she finished their cavity. I will compare this to the public healthcare dentist experience I have had before, where even if it is a deep hole and painful, they still do it without pain medicine.”* At the same time, another stated, “*I was nervous because the last dentist was really bad and spoke rudely. So, initially, while visiting the onsite dental office, I felt nervous and a little ashamed. But now I feel good and very comfortable; I have been going for six visits.”*

The fourth theme, Diet Influences on Oral Health, explores the influence of sugar intake across generations on oral health. Many participants (*n *= 18; 51.75%) reported frequent sugar ingestion. One participant mentioned, “*Everybody in the family likes sweets. We all eat candies at least once a day.”* One of the interviewees believed toothbrushing was the primary oral hygiene habit that could help mitigate the impact of sugar consumption. The participant expressed, “*If you eat too much of the sugary foods, then it's harmful to your teeth, but if you eat a few during the day, but then you brush your teeth afterward, then it will be fine.”* Some participants reported that they (*n *= 6; 23.12%) kept candies in their pockets while at work to gain a quick energy boost throughout the day. This was also described as a long-standing workplace tradition. Parents acknowledged feeding preschool-aged children snacks and beverages that contain significant amounts of sugar as a quick fix. One participant stated, “*Occasionally, I allow my children to have two servings of juice [including Pepsi and Coke]. When I notice that they drink the juice quickly, I think they are thirsty. In such cases, I say, ‘You can have another one if you want to.”*

The fifth and final theme, Self-reported Oral Health Status, explores self-reported oral hygiene habits and health problems. Over half (*n *= 19; 52.78%) of the participants reported experiencing pain related to their oral health. One respondent recalled their most recent dental pain experiences and stated, “*I couldn't ignore the constant, throbbing ache in my tooth that night. It was like a sharp reminder that dental pain is not something to be taken lightly.”* Some (*n *= 3; 8.33%) participants reported difficulty in chewing and only opted for a dental visit when they experienced acute pain. One participant expressed, “*I’ve never been good about taking my children to the dentist, and honestly, I don't go myself unless it hurts.”* Overall, several of the participants reported having oral health issues, including cavities (*n *= 6; 16.67%), commonly called “holes”, swelling in their oral cavities (*n *= 4; 11.11%), or bad breath (*n *= 5; 13.89%). Moreover, four (11.11%) participants reported replacing their toothbrushes less frequently than is recommended by the American Dental Association (3 months) (11).

### Extraoral photographic analysis of dental characteristics

Of the 36 participants in the subset, 28 individuals (77.78%) consented to participate in the extraoral photograph analysis, which analyzed the hard tissue, soft tissue, and smile characteristics.

#### Hard tissue analysis

**Figure 4:**
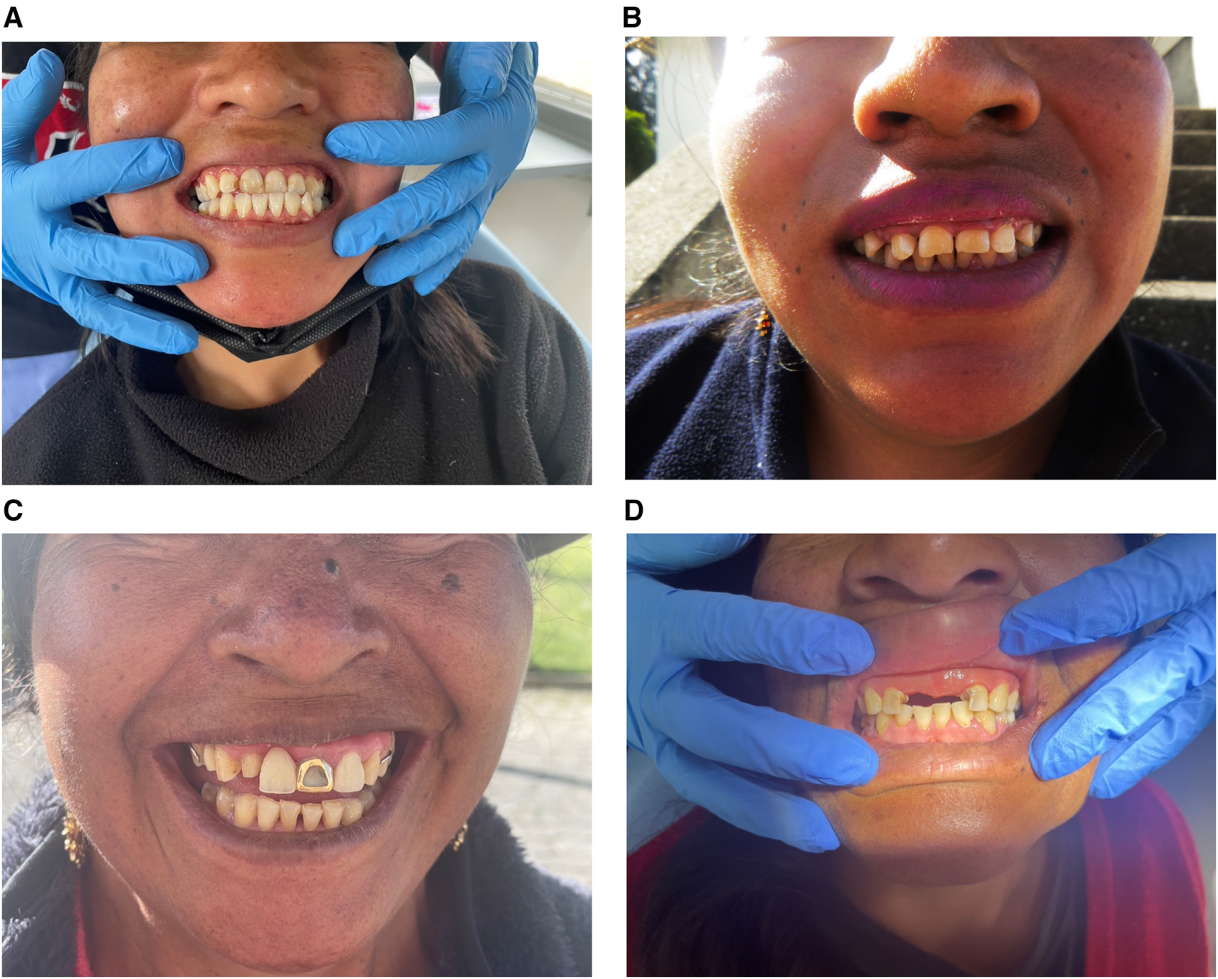
Extra-oral photographs, hard tissue analysis results. (**A**) Evidence of significant discoloration. (**B**) Evidence of attrition in anterior teeth. (**C**) Evidence of traditional dental decoration (golden crown). (**D**) Evidence of traditional dental mutilation.

Discoloration of teeth was observed in 89.29% (*n* = 25) of participants, which may have contributed to the considerable awkwardness observed in a relaxed smile (Figure 4A). Attrition was observed in more than three-quarters of participants (*n *= 22; 78.57%) (Figure 4B). Extra-oral photographs confirmed two styles of dental modifications (Figures 4C, D): dental ritual mutilations and dental decorations were mentioned as standard practices among older women. For example, a participant stated, “*I have a gold crown on my front tooth because I’m the priest of this community.”* Another participant shared, “*My front teeth were knocked off, not pulled to follow a religious custom when I was young.”*

#### Soft tissue analysis

**Figure 5:**
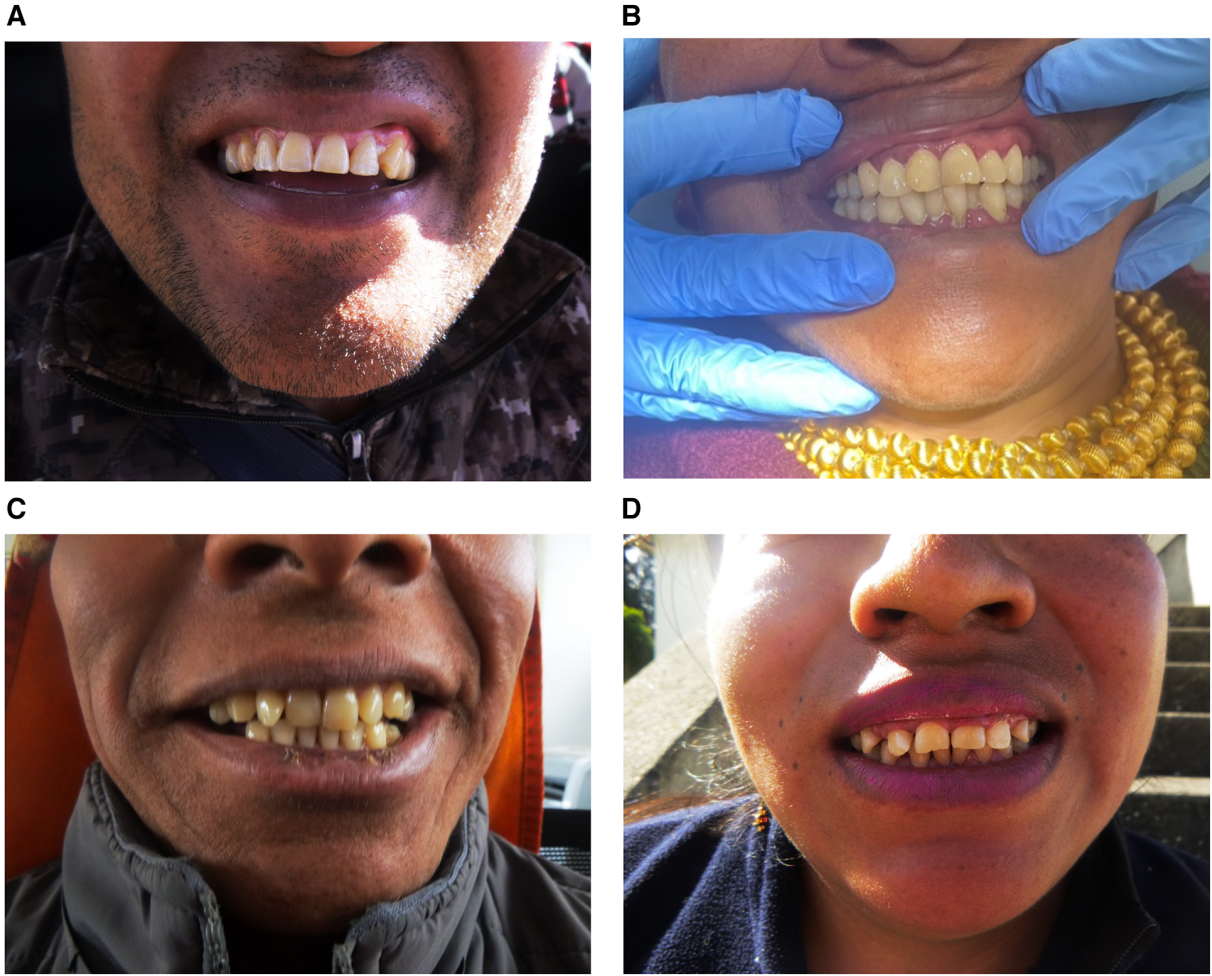
Extra-oral photographs, soft tissue analysis results. (**A**) Evidence of gingival swelling and gum recession. (**B**) Evidence of plaque-related gingival inflammation. (**C** and **D**) Evidence of lip dryness and discoloration.

Based on gingival redness and gingival swelling, gingival inflammation was observed in 11 (39.29%) participants (Figures 5A, B). Lip discoloration and dryness were noted in 9 (32.14%) and 14 (50%) participants, respectively (Figures 5C, D). Gingival recession was noted in 5 (17.86%) participants (Figure 5A).

#### Smile analysis

Anatomical assessment of smile characteristics revealed a fairly even distribution of high, medium, and low smiles across the study population (respectively: *n *= 11; 39.29%, *n *= 9; 32.14%, *n *= 8; 28.57%). A gummy smile was observed in one-quarter of the participants (*n *= 7; 25%). Malalignment of the lips (crooked smile) was also observed in one-quarter of participants (*n *= 7; 25%).

## Discussion

### Quantitative findings

Regarding oral hygiene practices, the majority (*n *= 466; 88.26%) reported brushing their teeth at least twice daily, indicating that participants prioritized oral hygiene in their daily routines. However, despite this, reported flossing rates were very low.

This discrepancy is consistent with previous literature, in which dental flossing as a self-performed preventive strategy is significantly low in LMICs such as Ecuador, where oral hygiene practices focus mainly on brushing (12). This phenomenon can be attributed to several factors, including socioeconomic inequities, lack of oral health awareness, and inadequate knowledge and education about oral health status (12). The cultural significance of these factors will be further elaborated upon in the thematic analysis discussion.

### Oral Health-Related Quality of Life (OHRQoL)

Although the sample reported a median OHRQoL score of only 4, participants' scores ranged from 3 to 14, demonstrating some participants' far reduced oral health-related quality of life. One potential explanation for this unexpectedly low score could partly be the Latino Paradox. The Latino Paradox is a phenomenon in which Latino ethnicity confers a protective effect on oral health outcomes, especially among immigrants to the United States (13, 14). It is possible, therefore, that this phenomenon is also being mirrored in the Ecuadorian population sampled in this study, thus resulting in lower OHRQoL scores than expected. Additionally, self-perceived and self-reported quality of life is a subjective measure influenced by numerous factors, including culture, age, social norms, and chronic disease status (15, 16), as well as social desirability and/or recall bias. Due to this subjectivity, participants' reported OHRQoL scores may not have accurately aligned with their clinical status or lived experiences.

Higher scores were found among older patients and those who reported poorer hygiene practices. This is in line with previously reported results in the literature, in which poor hygiene practices and older age have been associated with higher rates of oral conditions, which can influence an individual's quality of life (17–19).

Those with a higher educational level presented a poorer OHRQoL score. This finding differs from previous studies in both LMICs and high-income countries, where a lower educational level tends to be associated with a poorer OHRQoL (18, 20–22). No clear explanation for this could be identified from the current literature; however, we hypothesize that individuals with higher education may have been more acutely aware of how oral health impacts their quality of life or that they may have felt more comfortable sharing these effects with the interviewers. Further research should be done to elucidate potential reasons for this relationship.

### Qualitative findings

The first theme identified, “Oral Hygiene Routines & Habits,” described the oral hygiene practices of participants. Most participants reported regular tooth brushing as their primary oral hygiene practice; however, very few reported using dental floss regularly. The high rates of regular toothbrushing reflect positive behaviors within the sample; however, it has been found that self-reported toothbrushing practice may be inflated due to reporting bias, so results should be interpreted with caution (1). This trend of low flossing prevalence has been extensively reported, including in the United States (23, 24) and Indigenous communities in Ecuador (25). Interestingly, while studies in the United States show ranges of 20–30 percent of the population not flossing (23, 24), these rates have been reported as much higher in Indigenous Ecuadorian communities, in the range of about 90 percent (25), which is very comparable to our findings. These findings are relevant, as regular flossing has been found to prevent and decrease the prevalence and severity of plaque, gingivitis, and gingival inflammation associated with periodontal disease, as compared to brushing alone (26, 27). As such, it is important to explore the barriers to the use of dental floss and implement targeted solutions, as will be explored in the upcoming themes.

The second theme, Knowledge Acquisition & Education, demonstrated the need for early and consistent oral hygiene education, as family and institutional influences shape habits from a young age. Parental influence is critical in establishing positive oral hygiene routines from an early age (28, 29). Previous studies have found that maternal influence has an especially strong role in a child's development of oral health practices and knowledge (29). A positive correlation has been found between maternal knowledge about oral health practices and their children's oral health status (28, 29). Our results also suggested that the effects of peer and sibling modeling may impact the population. Previous literature supports this as it shows that siblings shape oral health practices and attitudes toward dental care (29). Proposed mechanisms for this include providing a safe space for learning, modeling hygiene practices, and acting as a role model for engaging or abstaining from oral health-compromising behaviors, such as smoking, drinking alcohol, or consuming sugary foods/drinks (29). Our results also identified dental providers as the strongest influence on dental health education; however, as will be elaborated upon in Theme 3, unfortunately, many barriers often prevent individuals from regularly attending the dentist. Overall, these results highlight the importance of early childhood education for developing proper oral hygiene routines and knowledge. As such, it would be beneficial to leave room for more programs that improve access to oral health education. Previous studies have found that oral health promotion and education in schools can successfully result in children having improved knowledge and understanding of dental hygiene tools and practices (30) and demonstrating better oral hygiene routines, and subsequently improved oral health status (31). However, factors such as affordability, access, and fear can limit the transmission of this education, so such barriers will be further explored in the following theme analysis.

The third theme, “Barriers to Oral Hygiene and Dental Care,” highlighted, among others, the challenges posed by the cost of hygiene products and professional dental care. These expenses are significant impediments to accessing much-needed oral health services. This aligns with the literature showing that cost prohibits adherence to health-promoting behaviors, especially among lower socioeconomic groups (1, 12, 18, 32). Fear of dental visits was identified as another barrier to seeking dental care. Similar findings have been well described in the literature (1, 12, 33, 34). Although not recurrently reported, some participants reported barriers such as transportation, accessibility, and being unsure of proper hygiene techniques. These barriers have also been widely reported in previous literature (12, 32, 35), emphasizing the importance of education campaigns as described in Theme 2.

Interestingly, worksite dental clinics were reported to help eliminate barriers to access, such as transportation, time away from work, and cost. Additionally, several participants receiving regular dental care with a consistent provider reported that consistent care helped them overcome the fear and anxiety of dental visits. Similar interventions have been used in various settings (35–37), demonstrating high feasibility, cost-effectiveness, and acceptance of the benefited population. These worksite clinics have been found to facilitate multi-visit treatments while decreasing absence from work, enable development of a trusting relationship with the dental provider, and allow for regular provision of oral health education (35–37).

The fourth theme, Diet Influences on Oral Health, identified the influence of sugar intake in this population. Consumption of sugary drinks and candies was identified to be common in the sample, as many participants reported using them for a convenient and quick energy boost, or for cultural or habitual practices. The trend of high sugar consumption, especially in sugar-sweetened beverages, has been well reported throughout Ecuador, with a monthly average consumption of sugar-sweetened beverages of about 5 liters per capita (38). In Ecuador's Indigenous communities, sugars and fermentable carbohydrates also make up a fundamental part of their diet (25). It has been reported that individuals in these communities often give up various protein sources in exchange for sugars or other carbohydrates (25). This trend is widespread across the country. This phenomenon is not unique to Ecuador and has been increasingly identified in other LMICs (1). It has been proposed that the affordability and availability of sugar, combined with the lobbying of global food and drinks industries to delay institution of public health advertising or health taxes, may contribute to this issue (1). Sugar is the most critical risk factor in the development of dental caries, and as such, dental agencies uniformly recommend limiting sugar consumption, especially in children (1, 39). To combat this trend, in 2014, Ecuador implemented a food labelling regulation consisting of traffic light imagery to indicate sugar levels in packaged food and drinks, and a tax on sugary foods was implemented in 2016 (38). Despite these efforts, however, our results indicate a need for additional interventions.

Moreover, we identified that almost half of the participants reported being regular users of smoking tobacco or vapes or being regular alcohol consumers (46% and 44%, respectively). Interestingly, previous studies in Indigenous Ecuador communities have found the majority of these individuals to report they do not smoke or drink as often, whether this is due to religious/cultural beliefs or simply under self-reporting (25). It is possible, therefore, that our findings are also under-representative of the accurate rates of tobacco or alcohol use in the sample or that our participants came from diverse backgrounds and, therefore, held differing beliefs about tobacco and alcohol use. Tobacco use has been associated with poorer oral health outcomes, including, for example, changes in the microflora, caries, periodontal disease, and lip and oral cancer (1, 40). In addition, alcohol use has been associated with changes to the oral microflora that in turn, contribute to the development of similar oral health conditions (1, 40). Like the case of the sugar industry, the tobacco and alcohol industries exploit the vulnerability of LMICs (1). Moving forward, more strict regulations should be implemented, including tailoring advertising to children, limiting retail sizes of sugary foods and drinks, and culturally tailored campaigns to spread awareness about lifestyle influences on oral health.

The fifth and final theme, Self-reported Oral Health Status, identified pain as one of the main symptoms experienced concerning their oral health. Unfortunately, the presence of pain often indicates a more advanced oral health disease (41), which may suggest that participants were not seeking dental care early enough, especially if the pain was a primary symptom of interest to them. Previous studies have found similar trends, in which participants associate a lack of pain with an absence of oral health issues and do not seek dental care until they can no longer bear the pain (37). This is problematic, as studies have found that oral pain alone does not reliably predict the presence of caries or periodontal disease and that regular dental visits are associated with better outcomes than symptomatic visiting patterns (42). Moreover, the finding that pain was commonly reported despite low OHRQoL scores further points to the subjectivity of self-reported oral health status, which has been extensively described previously (17–19, 25). Difficulty in chewing, cavities, inflammation, and bad breath were also commonly reported in the sample. Oral health education and programs are needed to help individuals understand the importance of regular dental care, oral hygiene practices, and preventing oral health problems.

### Extraoral photographic analysis of dental characteristics

The use of extraoral photograph analysis allowed for examining both dental and facial characteristics. EOP analysis has been used extensively in clinical dental practice to supplement dental diagnoses, and aid in planning treatments (43). However, to our knowledge, this study presents the first use of EOP analysis in an academic setting, as a tool to characterize the epidemiology of oral disease in a particular community, and to help contextualize the self-reported quality of life findings.

#### Hard tissue analysis

Discoloration of teeth was observed in most of the participants. This discoloration can be related to the prevalence of dental fluorosis in rural Ecuador (44, 45). Dental fluorosis is a condition characterized by discoloration of the enamel due to early exposure to excessive fluoride concentrations and can result in enamel pitting, which creates a site for bacterial accumulation and decay (44). Although numerous public health campaigns promote the addition of fluoride to water due to its beneficial effects at decreasing caries, excessively high concentrations, such as those identified in rural Ecuador communities, can be detrimental (25, 45). These findings highlight the need for further regulation of water safety testing and treatment.

Attrition was also observed in a high percentage of the population. The most common causes of dental attrition are bruxism, bite misalignment leading to tooth sensitivity, tooth fracture, and temporomandibular joint tenderness (46).

Dental ritual mutilations and dental decorations were also identified, especially among older women. During the interviews, individuals with such dental modifications described them as being related to cultural or traditional practices. Such trends have been previously described throughout numerous cultures and populations worldwide, since prehistoric times, with Ecuador being one of the major populations in which this has been identified (47, 48). Commonly cited cultural reasons for these modifications include religious leadership, self-identification, or as a status symbol (48). These findings indicate that additional research is needed to better understand the role of culture and religion on oral health status for this population and to develop culturally competent frameworks for assessing oral health.

### Soft tissue analysis

Soft tissue analysis revealed indicators for poor periodontal health (49), including the presence of gingival inflammation in approximately 39 percent of patients. Additionally, lip discoloration and dryness were noted in many participants. Lip discoloration can be caused by numerous factors, one of which is smoking (50), which was commonly reported during participant interviews.

#### Smile analysis

Smile Analysis unveiled that 25% of patients had a malalignment of the lips (crooked smile), and 25% of participants had a gummy smile. This indicates that there is an increased need for orthodontic and restorative treatment, as well as further evaluation of general oral health in this population.

Overall, this EOP analysis revealed that the participants had many more dental pathologies than they had described themselves during the interviews, in which they only described pain as a symptom. This indicates a need for patient education about the importance of regular, as opposed to symptomatic, dental visits to prevent and manage underlying pathologies.

### Strengths and limitations

Several strengths are noted in the present study, including a substantial sample size for mixed-methods descriptive investigation. The research team consisted of interdisciplinary professionals from clinical dentistry and public health. Additionally, using interpreters who spoke fluent English and Spanish permitted data collection from non–fluent or Spanish-speaking participants. Moreover, this research design allowed for collaboration with the community through knowledge translation and promotion, data quality control, and implementation of valid and reliable instrumentation, such as the OHRQoL tool.

As to this study's limitations, purposive sampling methods were used, as the participants were recruited based on their attendance to Novulis clinics and willingness to participate, resulting in a non-random sample, which may introduce selection bias and affect the generalizability of findings. Since the individuals who agreed to participate are likely different from those who chose not to participate, a degree of selection bias is acknowledged. Therefore, despite efforts to include rural and remote areas in the sample, it is possible that the sample is not fully representative of all such regions or the national population, and its conclusions should be interpreted cautiously. Moreover, the relatively short data collection period may have resulted in seasonal variations in oral health conditions or access to dental care, not being captured in the results. Additional research with more diverse populations within Ecuador and over the course of a longer time period would further validate an analysis of self-reported OHRQoL and provide a deeper understanding of how oral health conditions and related quality of life evolve over time, particularly in response to interventions.

Qualitative research often uses smaller samples, as did ours with only 36 participants in our subset. This could limit the depth and breadth of insights into the broader population's oral health experiences and challenges. Furthermore, qualitative analyses relying on thematic analysis may be subjective, despite efforts to ensure consistency through independent readings. Photographic analysis is observer-dependent and should not be used to diagnose and treat patients as it is meant to support comprehensive dental evaluation. This analysis should be matched with oral examination results, interviews, and self-reported OHRQoL for further conclusions. Clinical variables were not collected for the study, limiting the correlation power between clinical diagnosis and self-perceived oral health status.

## Conclusion

To our knowledge, this is the first mixed-methods study that explored oral hygiene practices and OHRQoL, as well as the role of cultural practices, of individuals receiving care through worksite dental clinics in Ecuador. Despite being preventable and non-communicable, oral health diseases pose a substantial global health burden, particularly for LMICs like Ecuador. Through interviews, our study presents the impacts of this burden at the individual level, among particularly vulnerable communities. We also explored potential factors contributing to this issue, including cultural beliefs and systemic barriers to accessing oral health care. The availability gap in oral care services and the fear of seeing a dentist have led to the persistence of prevalent oral health diseases among the Ecuadorian communities assessed in our study.

The study's findings underscore the need to advance oral health equity through multi-level interventions targeting infrastructure and oral health literacy. To achieve this, further research should be done to understand such vulnerable or underserved communities better and to develop culturally competent education and outreach programs to elevate their understanding and capacity around optimal hygiene practices. Such research should also be directed to inform higher-level policy interventions encompassing infrastructure, health literacy, and customized outreach campaigns to promote positive behaviors and advance oral health equity in underserved regions. Furthermore, future research and interventions should be carried out with the involvement of local community stakeholders, to ensure they are culturally sensitive and sustainable in the long-term.

## Data Availability

The raw data supporting the conclusions of this article will be made available by the authors, without undue reservation.
