# Supplementary material for: Assessing the impact of oral health disease on quality of life in Ecuador: a mixed-methods study
Source: Front Oral Health. 2024 Jul 18;5:1431726. doi: 10.3389/froh.2024.1431726 (PMC11291228; doi:10.3389/froh.2024.1431726)
Supplement: Supplementary file 1 [file Table1.docx]

**Supplementary Table 1.** Consent form

| **CONSENT & INFORMATION SHEET FOR RESEARCH STUDY**  **6-digit identifier:**   \|  \|  \|  \|  \|  \|  \|  \| \| --- \| --- \| --- \| --- \| --- \| --- \| --- \| | |
| --- | --- | --- | --- | --- | --- | --- | --- | --- |
| **STUDY TITLE:** Assessing the Impact of Oral Health Disease on Quality of Life in Ecuador: A Mixed-Methods Study | |
| **INTERVIEWER NAME:**  *Priyanka Gudsoorkar, Nupur Krishnan, Jasmin Benitez, Greg Krupa* | **PHONE NUMBER (24-hour Emergency Contact)**  *+1 (513)2387513* |

| **INTRODUCTION**  You are being asked to take part in a research study. Please read this paper carefully and ask questions about anything you do not understand.  **WHO IS DOING THIS RESEARCH STUDY?**  Priyanka Gudsoorkar, Director of Development at the Solidarity Dental Foundation, is the person in charge of this research study. There may be other people on the research team helping at different times during the study.  **WHAT IS THE PURPOSE OF THIS RESEARCH STUDY?**  The purpose of this research study is to, through mixed methods, better understand the oral hygiene status of individuals residing in Ecuador and to assess the effect of oral health disease on the oral health-related quality of life (OHRQoL) in our sample population.  **WHO WILL BE IN THIS RESEARCH STUDY?**  About 500 participants will take part in the quantitative portion of this study, while 30-40 people will take part in the qualitative portion of this study.  You may be in this study if you are 18 and older and reside in Ecuador.  **WHAT WILL YOU BE ASKED TO DO IN THIS RESEARCH STUDY AND HOW LONG WILL IT TAKE?**   - You will be asked to participate in a survey and an interview with the aid of an interpreter. - The screening survey will have demographic questions to collect age, sex, and area of residence. - The Oral Health Quality of Life (OHRQoL) questionnaire will be a three-question survey. - The interviews will take 45 - 60 min and be audio recorded. - At the end of the one-on-one interview (only), your picture focusing on the mouth region (only) will be collected. - No other identifying information will be collected.   **ARE THERE ANY RISKS TO BEING IN THIS RESEARCH STUDY?**  You are not expected to be exposed to any risk by being in this research study.  **ARE THERE ANY BENEFITS FROM BEING IN THIS RESEARCH STUDY?**  Because of being in this research, you might be introduced to the brushing technique, be made aware of the importance of brushing twice daily, and other optimal oral hygiene practices.  **WHAT WILL YOU GET BECAUSE OF BEING IN THIS RESEARCH STUDY?**  Each participant will receive a kit to help maintain daily oral hygiene comprising fluoridated toothpaste and toothbrush.  **DO YOU HAVE CHOICES ABOUT TAKING PART IN THIS RESEARCH STUDY?**  If you do not want to participate in this research study, you may not participate.  There is a place at the end of this paper to mark your choice.  **HOW WILL YOUR RESEARCH INFORMATION BE KEPT CONFIDENTIAL?**  The following procedure will be followed:   - de-identification of transcripts, photos, and recorded information, with individuals who participate in the study receiving a unique six-digit identification that will be used to maintain participant anonymity - use of substitutions for generic descriptions of other personal data - storage of all audio recorded files on a password-protected computer located in a private office accessed by key (only) - storage of all administered and completed paper surveys in a locked storage container located in a private office accessed by key (only) - each respondent will receive notification via informed consent that only aggregated results of this research will be published - The data from this research study may be published, but participants will not be identified by name. - Information that could identify you will be removed from the study data. After removal, the study data could be used for future research studies. The study data could also be given to another researcher for future studies. This may be done without getting additional permission from you. - Agents of the community partner Novulis and funding organization InterAmerican Development Bank (IDB) may inspect study records for audit or quality assurance purposes.   **WHAT ARE YOUR LEGAL RIGHTS IN THIS RESEARCH STUDY?**  Nothing in this consent form waives any legal rights you may have.  **WHAT IF YOU HAVE QUESTIONS ABOUT THIS RESEARCH STUDY?**  If you have any questions or concerns about this research study, you should contact  Priyanka Gudsoorkar email:  [priyanka@solidaritydental.org](mailto:priyanka@solidaritydental.org)  **DO YOU HAVE TO TAKE PART IN THIS RESEARCH STUDY?**  No one has to be in this research study.  Refusing to take part will NOT cause any penalty or loss of benefits that you would otherwise have.  You may start and then change your mind and stop at any time.  **HOW WILL THIS RESEARCH STUDY AFFECT YOUR LEGAL STATUS?**  Being in this study or refusing to be in this study will have NO EFFECT on your court case, probation, or parole.  You will NOT get in trouble for refusing.  You will NOT get special privileges if you agree.  BY TURNING IN YOUR COMPLETED SURVEY (or BY TAKING PART IN THESE ACTIVITIES), YOU INDICATE YOUR CONSENT FOR YOUR ANSWERS IN THIS RESEARCH STUDY.  By completing this survey and initialing the consent form, you voluntarily agree to participate in the research and that the information about this study has been satisfactorily explained to you. You understand you have the right to withdraw or discontinue participation at any time without penalty or loss of benefits to which you are otherwise entitled.  **PLEASE KEEP THIS INFORMATION SHEET FOR YOUR REFERENCE.** |
| --- |

**Supplementary Table 2.** Demographic questionnaire & OHRQoL measure

| **Demographic Questionnaire**  1. Age:  2. Sex:  3. Country of Origin:  4. Area of Residence:  5. Average Annual Household Income:  6. Education Level:  7. Brushing Frequency:  8. Flossing Frequency:  9. Tobacco Use:  10. Alcohol Use:  **6-digit identifier:**   \|  \|  \|  \|  \|  \|  \|  \| \| --- \| --- \| --- \| --- \| --- \| --- \| --- \|   **Oral Health Related Quality of Life (OHRQoL)**  **Dimensions Measured:** Daily activities, social activities, conversation  **Response format:** 5-point Likert scale “Always” to “Never”  1. Have problems with your teeth or mouth affected your daily life (e.g., going to school, going to work)?   \| Always \| Frequently \| Occasionally \| Rarely \| Never \| \| --- \| --- \| --- \| --- \| --- \| \| 5 \| 4 \| 3 \| 2 \| 1 \|    2. Have problems with your teeth or mouth affected your ability to socialize (e.g., playing outdoors, meeting with friends, going to see family)?   \| Always \| Frequently \| Occasionally \| Rarely \| Never \| \| --- \| --- \| --- \| --- \| --- \| \| 5 \| 4 \| 3 \| 2 \| 1 \|   3. Have problems with your teeth or mouth affected your ability to talk?   \| Always \| Frequently \| Occasionally \| Rarely \| Never \| \| --- \| --- \| --- \| --- \| --- \| \| 5 \| 4 \| 3 \| 2 \| 1 \| |
| --- | --- | --- | --- | --- | --- | --- | --- | --- | --- | --- | --- | --- | --- | --- | --- | --- | --- | --- | --- | --- | --- | --- | --- | --- | --- | --- | --- | --- | --- | --- | --- | --- | --- | --- | --- | --- | --- |

**Supplementary table 3. Semi-structured interview guide**

| Please state the six-digit identifying number assigned to you during the consent process.  **Background**  1. Can you please tell me about your family or the people in your household? How many children live in your household?  **Self-Oral Care**  2. Can you tell me about how you regularly clean your teeth/mouth?  a. How would you describe the toothbrush you use?  b. How would you describe the toothpaste you use?  c. Describe what alternatives/substitutes you use to toothpaste and/or toothbrush if you do not have either.  d. Do you use any of your fingers to clean your teeth?  e. Do you brush your teeth? If yes, how often? If not, why not?  f. If the answer to “e” is yes, what times of the day does brushing usually happen (morning, night, both, other times?) Why do you think these are the best times for you to brush?  g. If the answer to e is yes, how long do you brush at once?  h. Do you use any other product you think is good for oral hygiene?  i. How would you describe your brushing technique? (Round, up & down, front, and back)  j. At what age did you start brushing your teeth? Why?  k. Do you clean your tongue? If yes, how? If not, why not?  l. Where do you brush your teeth? Can you describe the location of the brushing area?  m. Describe the source of water that is available at the time of brushing.  n. How do you clean your toothbrush after use and store it?  o. How old is your present toothbrush? Usually, how often do you change your toothbrush?  p. How do you decide you have to change your toothbrush?  q. How often do you have to replace or buy a new toothpaste?  r. Do you consume sugary food/drinks? If yes, how many times each day?  s. Do you use tobacco? If yes, how many times each day, and how do you consume it?  t. Do you drink alcohol? If yes, how many drinks do you have per week?  **Family Oral Care**  3. Do all other adults in your household brush their teeth? If yes, how many times? If not, why not?  a. Do all adults in your household have their toothbrush?  b. Do any adults in your household share a toothbrush?  c. Do adults in your household use toothpaste similar to what you use? If not, how different is  their toothpaste?  d. How similar or different is the brushing technique for the other adults in your household?  **Question 4 and sub-questions can be excluded for adult-only households.**  4. How do you or your family members help maintain healthy teeth for child/children in the household?  a. Do children in your household have their toothbrushes? Do any of the children share a toothbrush?  b. Do children in your household use toothpaste similar to what you use? If not, how different is their toothpaste?  c. Do all children in your household brush their teeth? Do they brush by themselves? Do adults assist?  d. At what age did or do you expect a child to start brushing their teeth? Why?  e. Until what age do adults assist children in brushing their teeth?  f. How often do children brush their teeth? Why or why not do they brush that often?  g. How did your household’s children learn to brush their teeth?  **Narrative Routine**  5. Could you walk me through your toothbrushing sequence this morning? Is this what your morning brushing routine usually looks like? Why or why not?  **Oral health promotion/prevention subset of questions**  6. Would you make any changes to maintaining your oral health? Why or why not?  7. Would you like to share any concerns with brushing your teeth?  **Oral healthcare experience**  8. Describe your last experience at the dental office.  **COVID-19 related**  9. Could you consider any changes in your household’s oral hygiene routines and oral healthcare access during the COVID-19 pandemic? |
| --- |
